# Oropharyngeal cancer and human papillomavirus: a visualization based on bibliometric analysis and topic modeling

**DOI:** 10.3389/fmicb.2024.1387679

**Published:** 2024-05-29

**Authors:** Zhu Liu, Haixu Wang, Yang Xu, Hongming Wei, Yuchong Zhang, Huilei Dong

**Affiliations:** ^1^Department of Head and Neck Surgery, Cancer Hospital of China Medical University/Liaoning Cancer Hospital & Institute, Shenyang, China; ^2^Department of Abdominal Osteomalacia Radiotherapy, Cancer hospital of China Medical University/Liaoning Cancer hospital and Institute, Shenyang, China; ^3^Department of Medical Oncology, the First Hospital of China Medical University, Shenyang, China

**Keywords:** oropharyngeal cancer, HPV, treatment strategies, topic modeling, bibliometric analysis

## Abstract

**Objectives:**

The incidence of oropharyngeal cancer (OPC) is increasing. This study used bibliometric analysis and topic modeling to explore the research trends and advancements in this disease over the past 10 years, providing valuable insights to guide future investigations.

**Methods:**

7,355 English articles from 2013 to 2022 were retrieved from the Web of Science Core Collection for bibliometric analysis. Topic modeling was applied to 1,681 articles from high-impact journals, followed by an assessment of topic significance ranking (TSR). Medical Subject Headings (MeSH) terms were extracted using R and Python, followed by an analysis of the terms associated with each topic and on an annual basis. Additionally, genes were extracted and the number of genes appearing each year and the newly emerged genes were counted.

**Results:**

The bibliometric analysis suggested that the United States and several European countries hold pivotal positions in research. Current research is focused on refining treatments, staging and stratification. Topic modeling revealed 12 topics, emphasizing human papillomavirus (HPV) and side effect reduction. MeSH analysis revealed a growing emphasis on prognosis and quality of life. No new MeSH terms emerged after 2018, suggesting that the existing terms have covered most of the core concepts within the field of oropharyngeal cancers. Gene analysis identified TP53 and EGFR as the most extensively studied genes, with no novel genes discovered after 2019. However, CD69 and CXCL9 emerged as new genes of interest in 2019, reflecting recent research trends and directions.

**Conclusion:**

HPV-positive oropharyngeal cancer research, particularly treatment de-escalation, has gained significant attention. However, there are still challenges in diagnosis and treatment that need to be addressed. In the future, more research will focus on this issue, indicating that this field still holds potential as a research hotspot.

## 1 Introduction

Oropharyngeal cancer (OPC) is a common malignant tumor in the field of head and neck surgery, particularly in western countries, where its incidence is showing an upward trend (Day et al., 2020; Damgacioglu et al., 2022; Chaturvedi et al., 2023). In the past, OPC was thought to be associated with smoking and drinking. As a result, treatments for OPC have been similar to those for other squamous cell cancers of the head and neck.

However, with a deeper understanding of the role of human papillomavirus (HPV) in OPC, researchers realized that significant adjustments in diagnosis and treatment are needed. Studies show that HPV-positive OPC accounts for about 33%, is more common in men, and has a higher incidence in western regions (Carlander et al., 2021; Lechner et al., 2022). In addition, HPV-positive OPC differs from previous treatment categories, has a better prognosis, and is now considered a specific disease entity. With the increasing rate of HPV infection, this has led to a further decline in OPC mortality and has also focused attention on HPV screening and prophylactic vaccination (Chaturvedi et al., 2011).

While HPV-positive OPC holds promising prospects, treatment strategies remain debated. Even with a good prognosis, concerns rise over side effects like dysphagia, speech issues, and social challenges. Research now aims to balance efficacy with fewer side effects. Some explore treatment de-escalation, but conclusive results are pending. In addition, treatment outcomes for HPV-negative OPC are less favorable than HPV-positive cases, necessitating further research and enhancement.

To address the above issues, a large number of research papers are constantly being produced, making it challenging for researchers to sift through the vast amount of literature to identify the latest research advances. While there are a few bibliometric studies on head and neck tumors, there is limited research specifically focused on OPC. To address this problem, we used bibliometric and topic modeling to comprehensively analyze the relevant literature. This approach combines the quantity and quality of the literature, providing us with a panoramic view of research in the field, helping us to systematically sort through the topics related to OPC and highlighting those areas of research that may have been overlooked but have significant potential value.

## 2 Materials and methods

**Figure 1:**
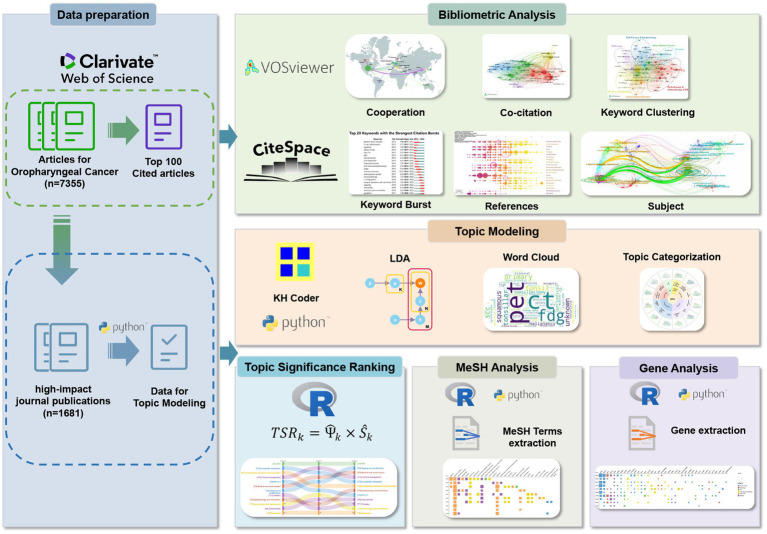
Overall flow of the study.

Our study integrated several steps: data acquisition, bibliometric analysis, topic modeling, topic significance ranking (TSR), analysis of Medical Subject Headings (MeSH) terms and genes. The detailed process for each stage is presented in Figure 1.

### 2.1 Data acquisition

We collected English articles related to OPC from the Web of Science Core Collection (WOSCC) Database from 2013 to 2022, detailed in Supplementary Table 1. We also downloaded the top 100 cited articles for additional bibliometric analysis.

### 2.2 Bibliometric analysis

We analyzed collaboration, co-citation, and co-occurrence using VOSviewer, enhancing visualizations with Scimago Graphica. Citespace has significant advantages in keyword analysis, subject analysis, and reference analysis, which can be used to generate burst analysis of keywords, dual-map overlays of journals, and timeline analysis of references.

### 2.3 Topic modeling

**Figure 2:**
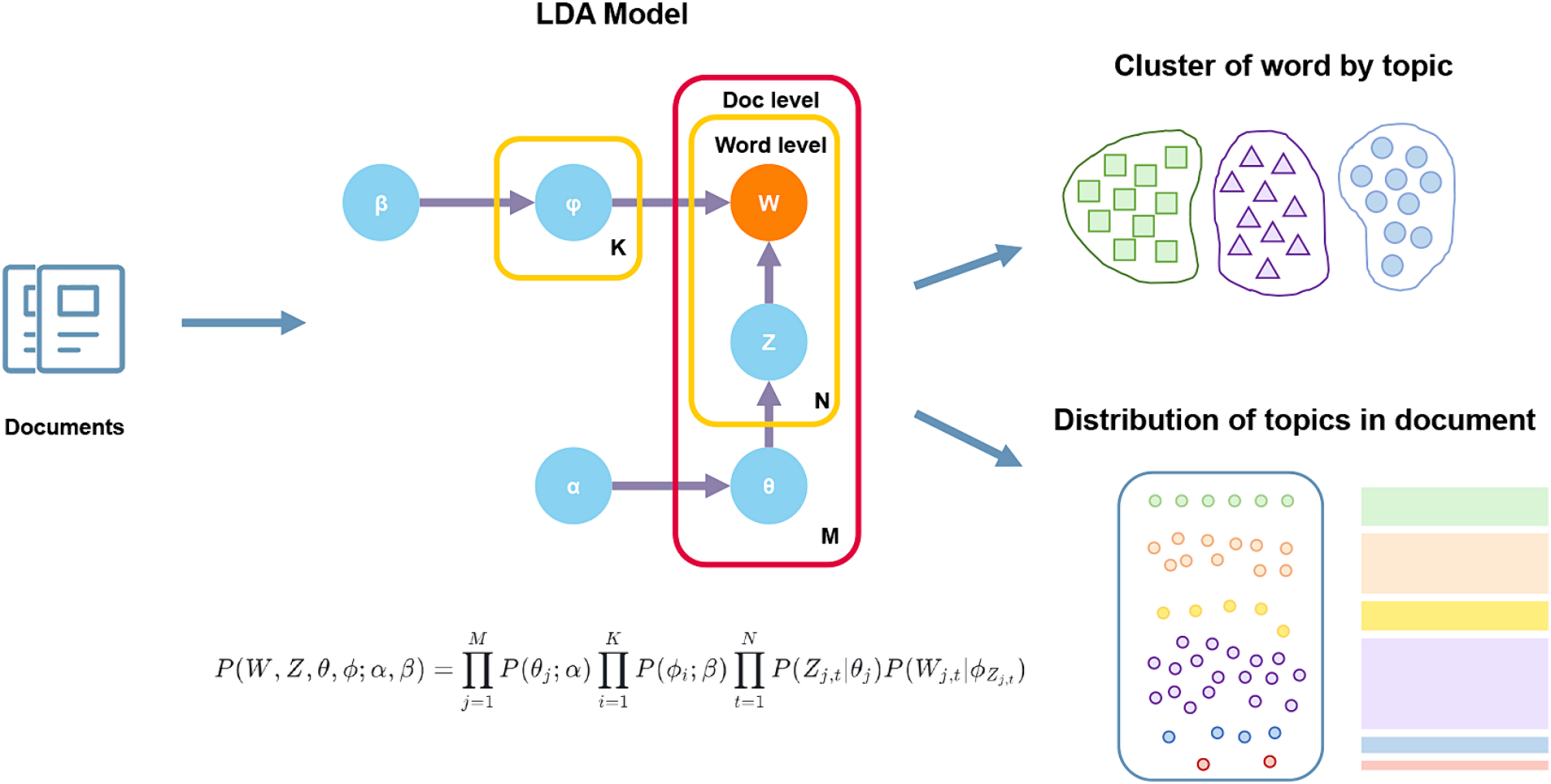
Schematic diagram of LDA principles. α, β, hyperparameters of the model; φ, word distribution of topics; θ, topic distribution of documents; Z, topic distribution corresponding to words; W, all words in the documents; K, total number of topics; N, number of words in the document; M, total number of documents. $\theta_{j}\text{,}$ represents the topic distribution of document *j*; $\phi_{i}$, represents the word distribution of topic *i*; $Z_{j,t}$, represents the topic assignment of the *t*-th word in document *j*.

Considering journal quality variations, we retrieved high-impact journal publications using the query in Supplementary Table 1, analyzing titles and abstracts for topic modeling. Topic modeling is an advanced method for unsupervised clustering of unstructured text, capable of extracting hidden topics from vast document collections without relying on manual annotation. This technique plays a crucial role in interpreting and summarizing large volumes of text data. Unlike bibliometrics, which primarily focuses on clustering keywords and references, topic modeling delves deeper into text content, revealing more profound thematic layers. Latent Dirichlet Allocation (LDA), the most widely used topic modeling technique, assigns a topic to each word in a document and continuously refines the probability distribution of words to topics and topics to documents, effectively grouping publications with similar content based on the similarity in the usage of words in their abstracts. The specific principles underlying this approach are illustrated in Figure 2.

In this study, we utilized KHcoder software for LDA analysis. Data preprocessing is the first step in the analysis, which includes:

1. Removing noise from the text, such as special characters and punctuation;
2. Stemming, to standardize various word forms;
3. Checking tokenization, recognize words that should be grouped into a phrase, rather than individual words;
4. Removing stop words, such as common but insignificant words like “and,” “or,” “that.”

Additionally, we removed words that frequently appear but do not substantively aid in topic analysis, as well as words that occur too infrequently.

After data preprocessing, we used ldatuning to determine the optimal number of topics in the LDA model using a series of statistical metrics. This tool simplifies the selection process by identifying the minimum or maximum values of metrics, where achieving the lowest possible values for the metrics from Arun2010 and CaoJuan2009 and the highest possible values for those from Deveaud2014 and Griffiths2004 is preferable.

Based on the number of topics determined by ldatuning, we conducted topic clustering and identified keywords associated with each topic. We used Python to generate word clouds for visualization of these keywords, and based on these, we named each topic, ultimately categorizing them into six categories.

### 2.4 Topic significance ranking (TSR)

Although LDA can reveal topics within text data, the importance of these topics is not always equal. To delve deeper into the topics we identified and their trends over time, we divided the data into three periods based on a time series: 2013 to 2015, 2016 to 2018, and 2019 to 2022. This division allows us to track changes in topic significance within each period and chart the developmental trajectory of the field over time. TSR (AlSumait et al., 2009) serves as an unsupervised analytical tool that evaluates the inferred topics in a Probabilistic Topic Models (PTM) through unsupervised inspection, employing a 4-phase Weighted Combination decision strategy to assess their semantic importance. TSR integrates multi-criteria metric information, using a ‘Weighted Linear Combination’ (WLC) decision-making strategy, which has been widely applied in multi-criteria decision analysis (Bouyssou et al., 2006). The formula is given as Equation (1):


(1) $$A_{k}=\sum_{m=1}^{N_{m}}\Psi_{m}S_{m,k}$$

$N_{m}$ represents the number of measurement criteria, $\Psi_{m}$ denotes the weight of each criterion, and $S_{m,k}$ represents the score of the k-th topic relative to the m-th metric.

Step One: standardization procedures. TSR employs a four-stage weighted composite method, using calculated measurement results to construct scores $S_{m,k}$ and weights of different criteria $\Psi_{m}\text{,}$ requiring two standardized processes. The first involves recalibrating each score relative to the total scores across all topics, as shown in Equation (2). The second type utilizes the range of scores with the minimum and maximum values as standardization scaling points, as shown in Equation (3).


(2) $$\overset{´}{C}1_{k}^{m}=C_{k}^{m}\times \frac{\sum_{j=1,j\neq k}^{k}C_{j}^{m}}{\sum_{j=1}^{k}C_{j}^{m}}$$

(3) $$\overset{´}{C}2_{k}^{m}=\frac{C_{k}^{m}-C_{\text{min}}^{m}}{C_{\text{max}}^{m}-C_{\text{min}}^{m}}$$

$C_{\text{min}}^{\text{m}}$ and $C_{\text{max}}^{m}$ are the minimum and maximum measurement values under criterion C for measuring m, $m\in \left\{KL,COR,COS\right\}$, where KL divergence, correlation coefficient (COR), and cosine dissimilarity (COS) are the three metrics used to measure distance.

Step Two: Intra-criterion weighted linear combination. To integrate different criteria and obtain an overall score, WLC is used to calculate the standardized score of each topic under a specific criterion. Under criterion C, the WLC score for a given topic K is the average of scores from the three distance metrics, as shown in Equation (4):


(4) $$S_{k}^{C}=\frac{\overset{´}{C}{\;}_{k}^{KL}+\overset{´}{C}{\;}_{k}^{COR}+\overset{´}{C}{\;}_{k}^{COS}}{3}$$

Step Three: Inter-criterion Weighted Combination. The scores from each criterion are integrated using a Weighted Combination (WC) approach, employing two methods for assigning weights to the scores. The first method, as shown in Equation (5), is based on weighting by the scores of the background criteria. ${\;}_{\Psi {´}_{u}}$ and ${\;}_{\Psi {´}_{v}}$ represent the weights in the scores of Uniformity and Vacuousness criteria, respectively, while $\hat{S}1_{k}^{b}$ denotes the weight of the topic background. The second method is based on standardization by the score range, as shown in Equation (6):


(5) $${\hat{S}}_{k}=\hat{S}1_{k}^{b}\left({\overset{´}{\Psi }}_{u}S1_{k}^{u},+{\overset{´}{\Psi }}_{v}S1_{k}^{v}\right)$$

(6) $${\overset{⌢}{\Psi }}_{k}=\Psi_{u}S2_{k}^{u}+\Psi_{v}S2_{k}^{v}+\Psi_{b}S2_{k}^{b}$$

Step Four: Topic Significance Score. The overall score of each topic, obtained using Equation (5), is multiplied by the normalized weights derived from Equation (6). The final calculation of topic significance is shown in Equation (7):


(7) $$TSR_{k}={\overset{⌢}{\Psi }}_{k}\times {\overset{⌢}{S}}_{k}$$

### 2.5 MeSH and gene analysis

The analysis was expanded to include MeSH terms and genes, which revealed some notable findings. For MeSH terms, we first compiled PMIDs corresponding to the documents of each topic, organized the data using Python, and used the RISmed package in R to extract MeSH terms, remove duplicates, and calculate term frequency. This process identified the primary MeSH terms for each topic and their frequency of occurrence. In terms of data preprocessing, we used the plyr package to organize the data, retaining only those MeSH terms that appeared more than five times and exhibited significant differences across topics. Using the pheatmap package, we generated heatmaps to visually display the distribution of MeSH terms for each topic.

For the annual analysis of MeSH terms, we grouped articles by publication year and collected corresponding PMIDs. We also used the RISmed and dplyr packages to extract and tabulate MeSH terms for each year, subsequently plotting terms that appeared at least six times. We matched these terms with the corresponding topics and their six categories, annotating them with different colors. Additionally, we identified MeSH terms newly emerged each year.

To extract genes from text, we employed the Term Frequency-Inverse Document Frequency (TF-IDF) technique to preprocess the text, removing redundant information and preserving key content. Subsequently, we utilized Python to identify genes in each text segment and perform frequency analysis on the extracted genes, and used R to calculate the topics corresponding to the genes. We only retained genes that appear two or more times and show significant differences between different topics.

## 3 Results

### 3.1 Bibliometric analysis

**Figure 3:**
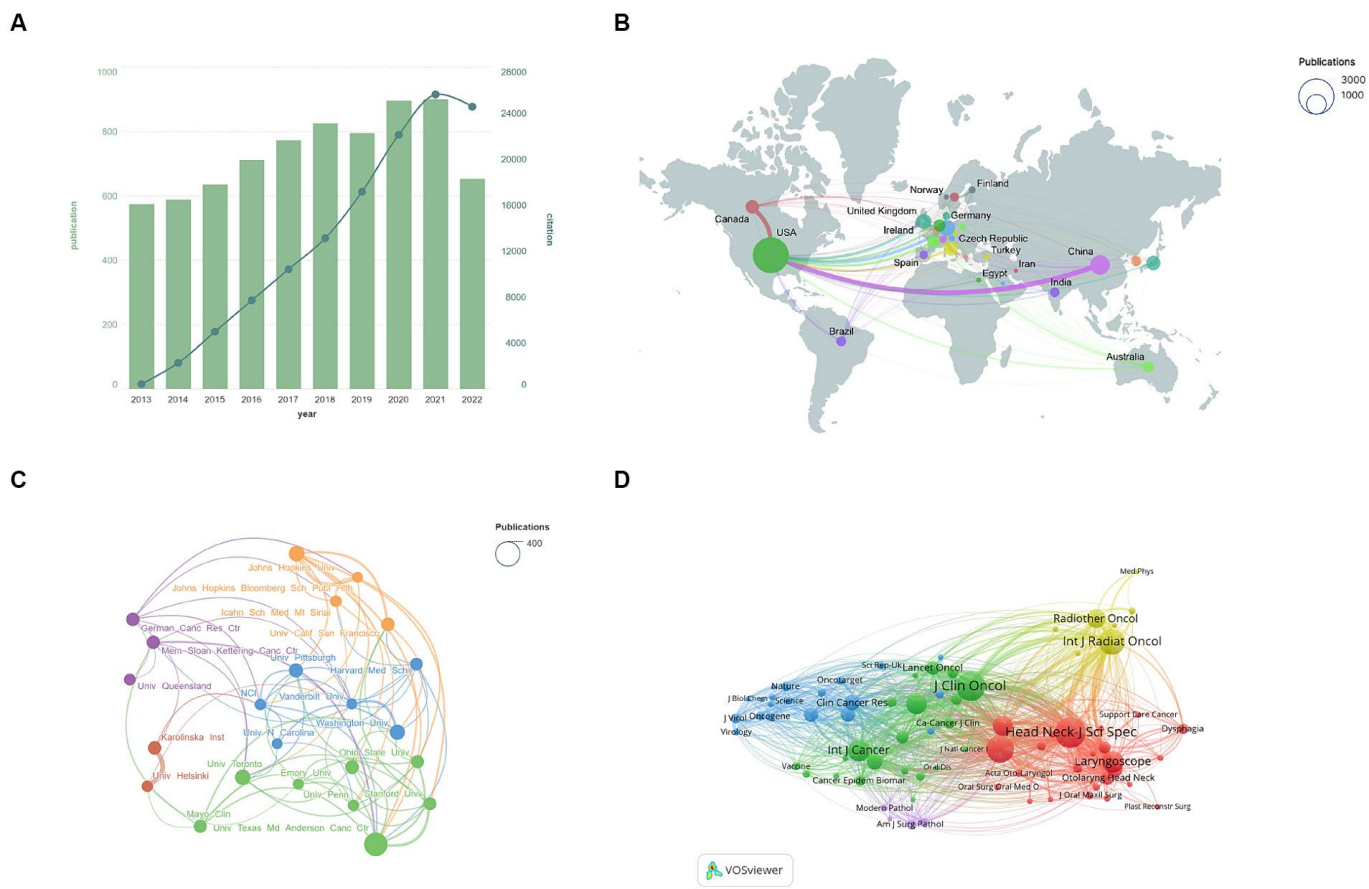
**(A)** Annual publication and citation trends, with bar graphs representing the number of publications and line graphs indicating citations. **(B)** Map of international collaborations. Circle size reflects publication volume, while line thickness indicates collaboration strength. **(C)** Institutional collaboration network. Circle size denotes publication count, and line thickness represents the intensity of collaboration. **(D)** Journal co-citation network.

A total of 7,355 articles were included in the analysis. Figure 3A reveals a modest rise in publications and citations over the decade. The data shows the United States and China as leading publishers with strong collaboration, while the U.S. has more extensive ties with Europe (Figure 3B). Table 1 lists the publication volumes of each country in the total literature and the top 100 most cited articles. It is noteworthy that European and American countries rank highly in terms of publication volumes of highly cited articles. Considering the higher number of HPV-related OPC cases and the higher prevalence of HPV vaccination in the European and American regions (Bruni et al., 2021; Carlander et al., 2021), this may indicate a greater investment in related research by these countries.

**Table 1:** Top 10 countries by publication count.

Rank	Country	Publications	Avg. citations	Country*	Publications*
1	USA	2,879	26.2167	USA	71
2	China	847	13.7556	Canada	21
3	Germany	534	22.1442	Netherlands	13
4	United Kingdom	512	24.1094	France	11
5	Japan	457	12.3698	United Kingdom	11
6	Italy	435	17.8161	Spain	8
7	Canada	406	35.6478	Germany	7
8	Netherlands	335	33.1493	Belgium	6
9	France	329	32.3313	Denmark	6
10	Australia	273	19.8095	Italy	5

*Top 100 citation.

Figure 3C and Table 2 highlight publication volumes and collaborations between institutions. The University of Texas MD Anderson leads in both aspects. The Karolinska Institute and the University of Helsinki collaborate closely. Interestingly, U.S. institutions are prevalent in top total and most cited publications.

**Table 2:** Top 10 institutions by publication count.

Rank	Institution	Publications	Avg. citations	Institution*	Publications*
1	Univ Texas Md Anderson Canc Ctr	330	38.7515	Univ Texas Md Anderson Canc Ctr	19
2	Johns Hopkins Univ	150	42.74	Ohio State Univ	13
3	Univ Toronto	142	48.5352	Univ Toronto	11
4	Washington Univ	138	34.1159	Stanford Univ	10
5	Univ Pittsburgh	118	36.9576	Johns Hopkins Univ	9
6	Univ Michigan	113	33.3451	Univ Calif San Francisco	9
7	Univ Calif San Francisco	111	38.8829	NCI	8
8	Karolinska Inst	109	18.2844	H Lee Moffitt Canc Ctr & Res Inst	6
9	German Canc Res Ctr	108	27.5926	Harvard Univ	6
10	Mem Sloan Kettering Canc Ctr	105	51.2095	Mem Sloan Kettering Canc Ctr	6

*Top 100 citation.

Journal co-citation analysis reveals latent connections among journals and quantifies their interconnections. In Figure 3D, the co-citation network within the field is displayed. The red cluster represents specialized journals in head and neck tumors, yellow and green clusters encompass oncology journals, the blue cluster contains general journals, and the purple highlights pathology-related publications. This diagram shows that research is focused in specialized journals and closely linked with oncology, with less interaction with general journals.

**Figure 4:**
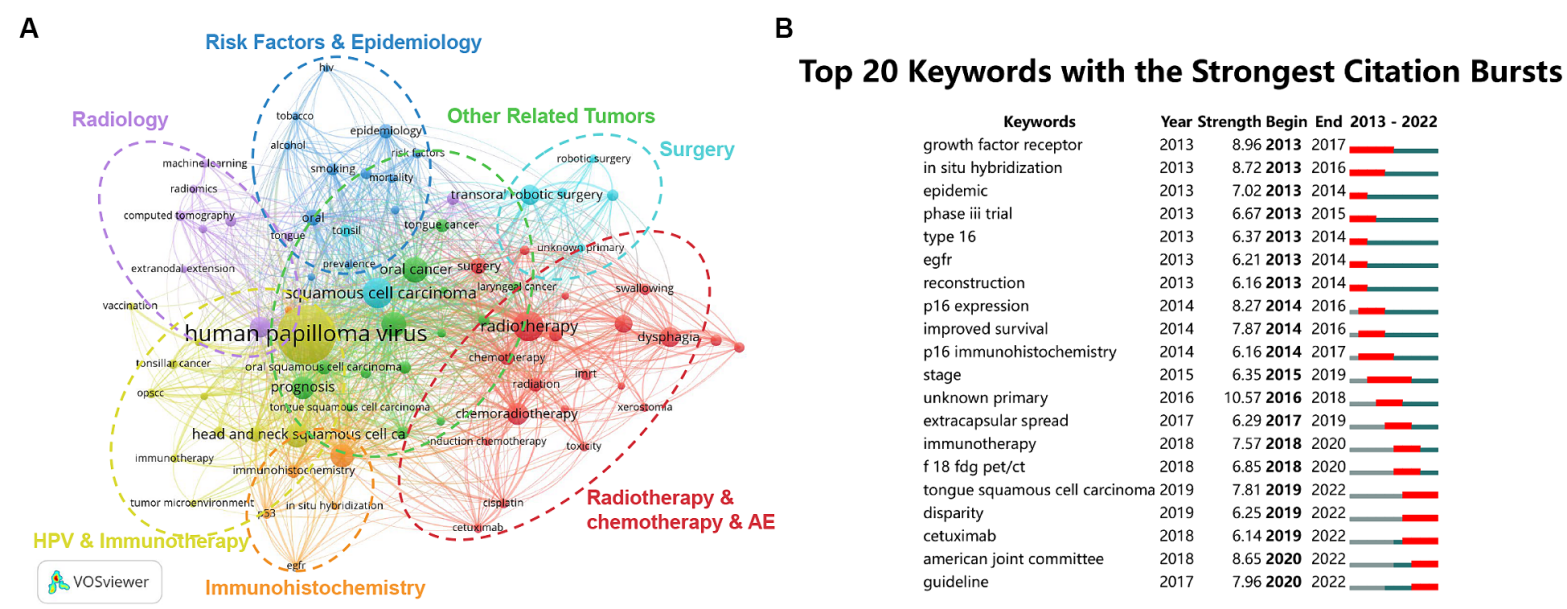
Keyword analysis. **(A)** Keyword co-occurrence network. The deep blue cluster highlights risk factors and epidemiology, while the purple cluster focuses on imaging studies. The light blue cluster emphasizes advancements in the surgical field, and the red cluster closely follows radiotherapy, chemotherapy, and their related adverse effects. The orange cluster focuses on immunohistochemical techniques, and the yellow cluster covers studies related to HPV and immunotherapy. The green cluster includes other tumors closely related to oropharyngeal cancer. **(B)** Top 20 emerging keywords. Red lines indicate the start and end years of the emergence. AE, adverse effects.

Figure 4 displays the results of the keyword analysis. Figure 4A shows a keyword co-occurrence analysis, highlighting the main research topics in the field: epidemiology and related risk factors; various diagnostic techniques, including imaging and immunohistochemistry; and diverse treatment approaches such as surgery, radiation therapy, chemotherapy, and immunotherapy, which also encompass studies related to HPV. The positioning of HPV is particularly prominent, indicating its central role in the research field and underscoring its significance and pivotal role in current scientific inquiries. The results of the keyword burst analysis are shown in Figure 4B, where some meaningless keywords have been removed. The hot research keywords of 2022 reflect further exploration into improving treatment methods (Cetuximab) and deeper studies on staging and stratification (American Joint Committee, guideline).

**Figure 5:**
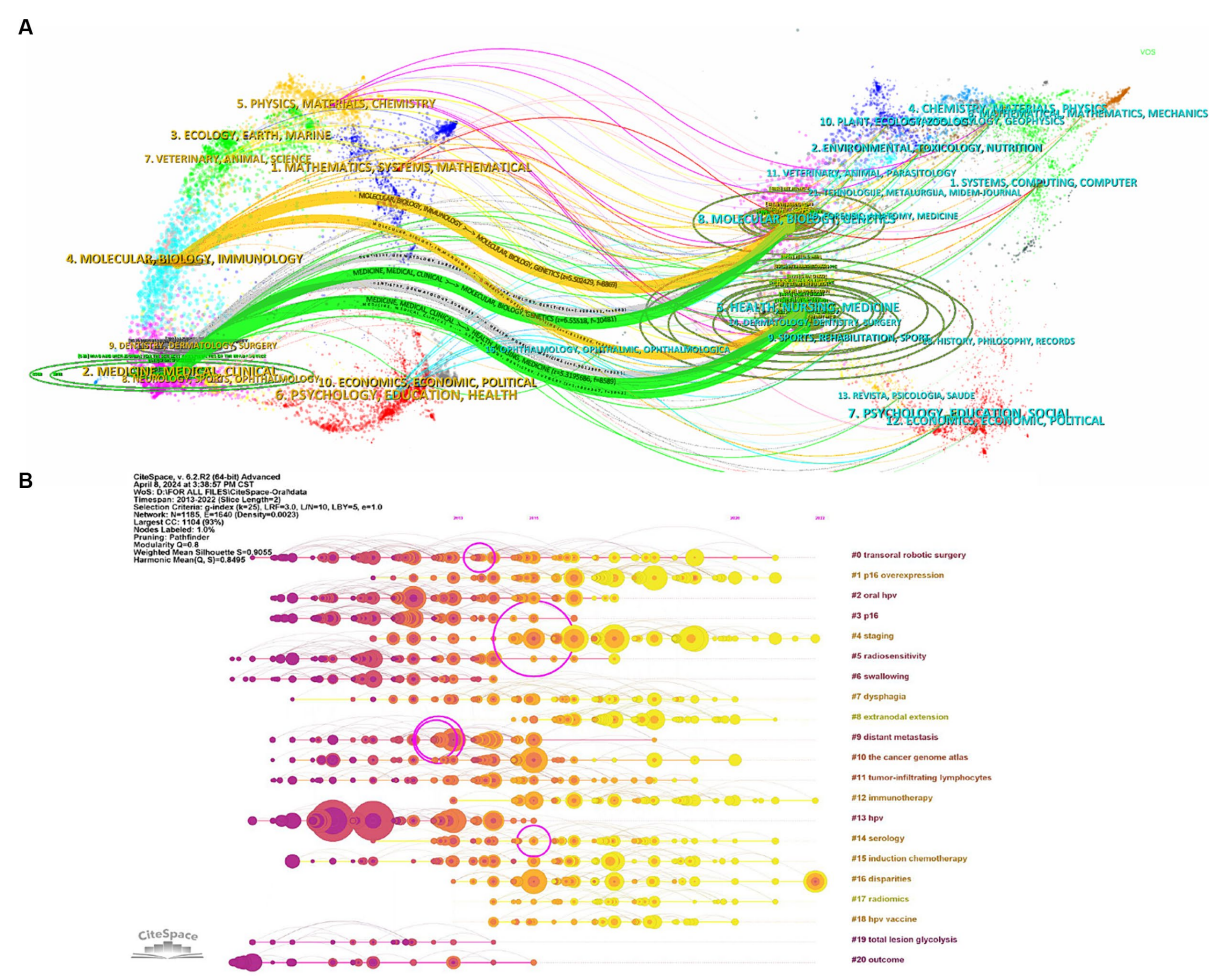
**(A)** Dual-map overlay. The left side shows citing journals, and the right side shows cited journals. **(B)** Reference timeline chart, where elements closer to the right represent recent research, reflecting the latest research trends and advancements within the field.

Subject analysis and reference evaluation are key tools for gaining insights into the dynamics of academic fields. In Figure 5A, we can observe the citation flows between journals, where journals on the right side of the figure are cited by journals on the left. Specifically, papers published in journals related to molecular/ biology/ genetics, health/ nursing/ medicine, and dermatology/ dentistry/ surgery are primarily cited by those in molecular/ biology/ immunology and medicine/ medical/ clinical. This pattern indicates a research focus leaning toward the integration of basic science and clinical practice, with relatively less cross-disciplinary interaction. Figure 5B, which displays a timeline of references, reveals shifts in research focus, with recent studies particularly concentrated in areas like “#16 disparities,” “#4 staging” and “#12 immunotherapy.” This highlights that current research focuses primarily on the efficacy and survival disparities in OPC patients, the staging of OPC, and immunotherapy.

### 3.2 Topic modeling

Bibliometrics analysis relies on information from various sources such as countries, authors, journals, keywords, and references, providing us with a preliminary framework regarding the historical development and trends of scientific research. To delve deeper into the historical context of the field and predict its future development, we have employed topic modeling techniques for extended analysis. Bibliometric analysis focuses on explicit quantitative indicators to evaluate publishing trends and collaborative networks, whereas topic modeling explores latent topics by analyzing the text in article titles and abstracts. Topic modeling enables the processing of large-scale data, extracting key concepts and ideas from the text, which guide the direction of subsequent research.

**Figure 6:**
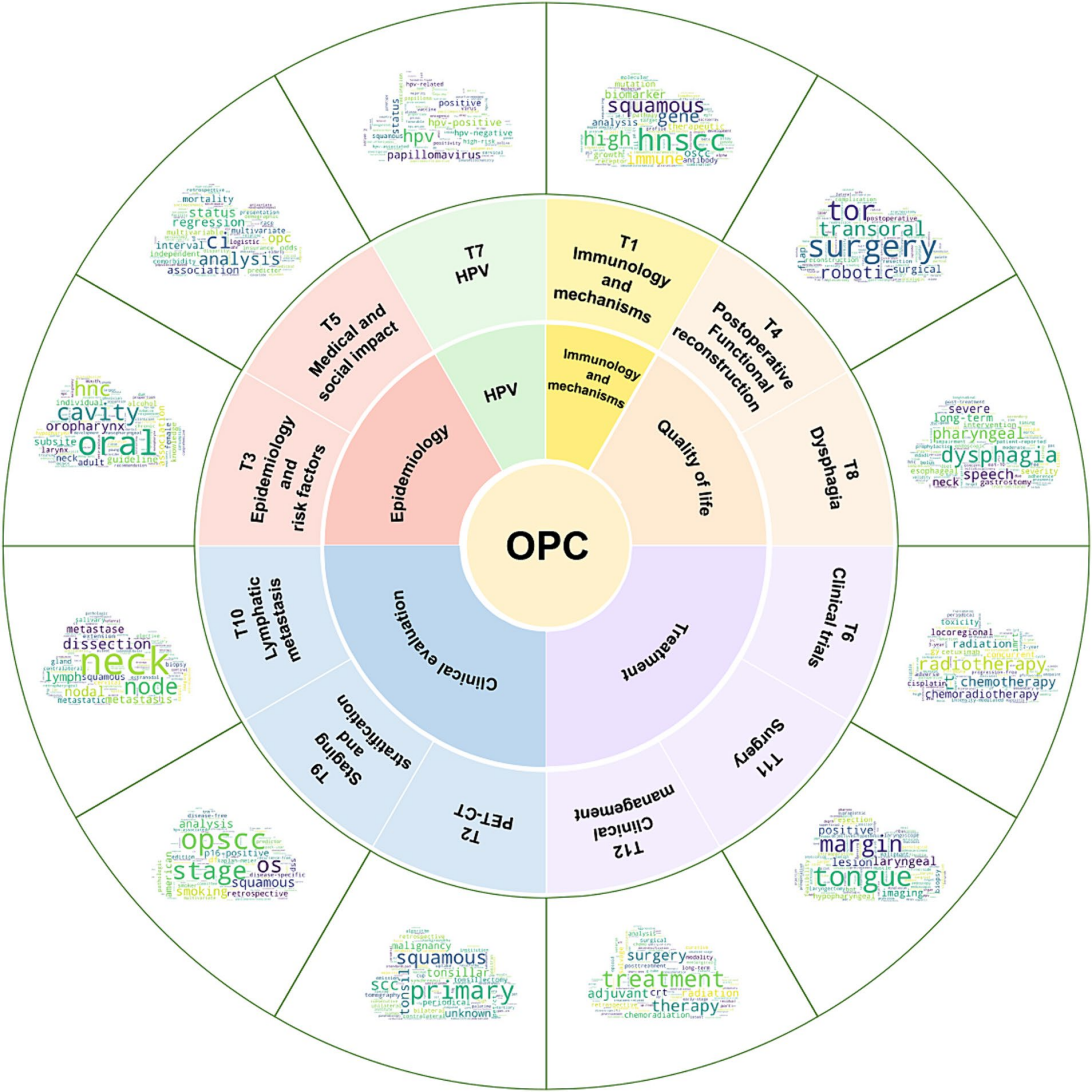
Topic identification and categorization of these topics. The outermost word cloud displays the keywords for each topic, where larger font sizes signify greater significance of the keywords within their respective topics. The naming of the topics (the layer below the word cloud) is based on these keywords, and the 12 topics are manually subdivided into six categories (inner circle): HPV, Epidemiology, Clinical evaluation, Treatment, Quality of life, Immunology and mechanisms. OPC, Oropharyngeal Cancer.

From 1,681 papers published in selectively high-impact journals, we identified 12 research topics based on the results from ldatuning (as shown in Supplementary Figure 1) and an overall assessment of the knowledge and volume of literature in related fields. To visually present the key content of these topics, we constructed a series of keyword clouds (see Figure 6). After manual review, we named each topic based on these keywords and ultimately categorized them into six categories: HPV, Epidemiology, Clinical evaluation, Treatment, Quality of life, Immunology and mechanisms.

### 3.3 TSR

**Figure 7:**
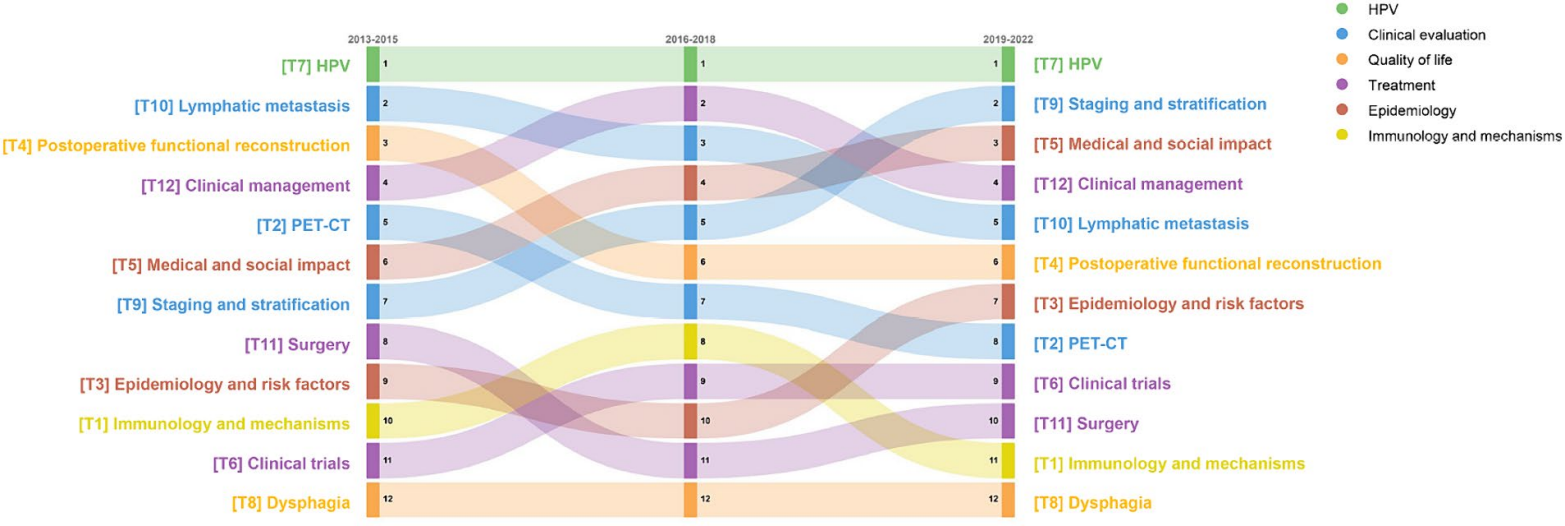
TSR changes over three time periods. Nodes higher up indicate a higher TSR ranking, signifying greater topic importance. Colors denote the category to which the topic belongs.

TSR results in Figure 7 rank the significance of each topic. Due to the pivotal role of HPV in OPC evaluation and treatment, T7 “HPV” consistently stands out in research. Variability among OPC patients combined with updated guidelines has heightened the importance of T9 “staging and stratification.” Furthermore, the rising relevance of research on the medical and social impact of OPC is evident, leading to a significant increase in T5.

### 3.4 MeSH analysis

**Figure 8:**
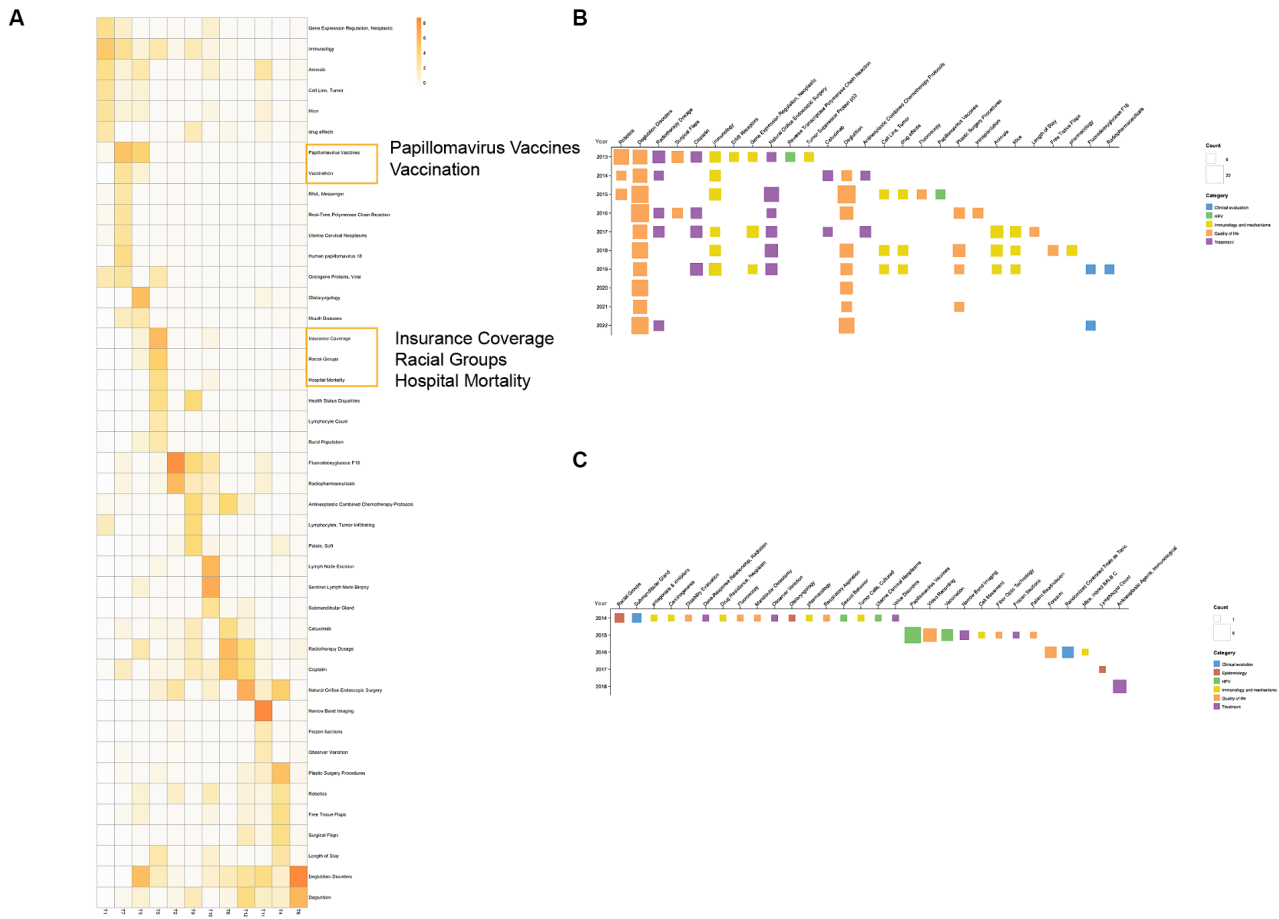
MeSH term analysis. **(A)** MeSH term-topic heatmap. The darker the color, the higher the relevance of the MeSH term to the topic. For easier observation, we have arranged topics from the same category in adjacent positions and enlarged the display of some MeSH terms. **(B)** High-frequency (frequency ≥ 6 times) MeSH terms annually. Based on the calculated topics corresponding to each MeSH term, the topics are mapped to six major categories, and they are color-coded according to their classification. **(C)** Newly emerged MeSH terms each year.

Figure 8A displays the MeSH term heatmaps for all topics, offering insights into their medical content. In T7, terms like “papillomavirus vaccine” suggest at an HPV link, while in T5, “insurance coverage” and “hospital mortality” suggest medical and social contexts.

Figure 8B presents MeSH terms appearing six or more times yearly, colored by topic. The “prognosis” category, with terms like “Deglutition Disorders,” is most frequent, highlighting a focus on prognosis and quality of life. Figure 8C shows yearly emerging MeSH terms, regardless of whether they were high-frequency MeSH terms or not. It’s important to note that our literature inclusion began in 2013, so the MeSH terms listed as newly emerged in 2013 may have actually existed earlier. Additionally, due to the abundance of MeSH terms, we started listing newly emerged MeSH terms from 2014 onwards. It can be observed that there are very few new MeSH terms in this field, and no new MeSH terms appear after 2018. This may indicate that most of the core concepts and topics in OPC are already covered by existing MeSH terms. It may also imply that research in this field has reached a certain bottleneck, with no significant new developments or breakthroughs. Therefore, current research may focus more on in-depth analysis of existing diagnostic, treatment and prognostic factors, rather than the introduction of new concepts or terms.

### 3.5 Gene analysis

**Figure 9:**
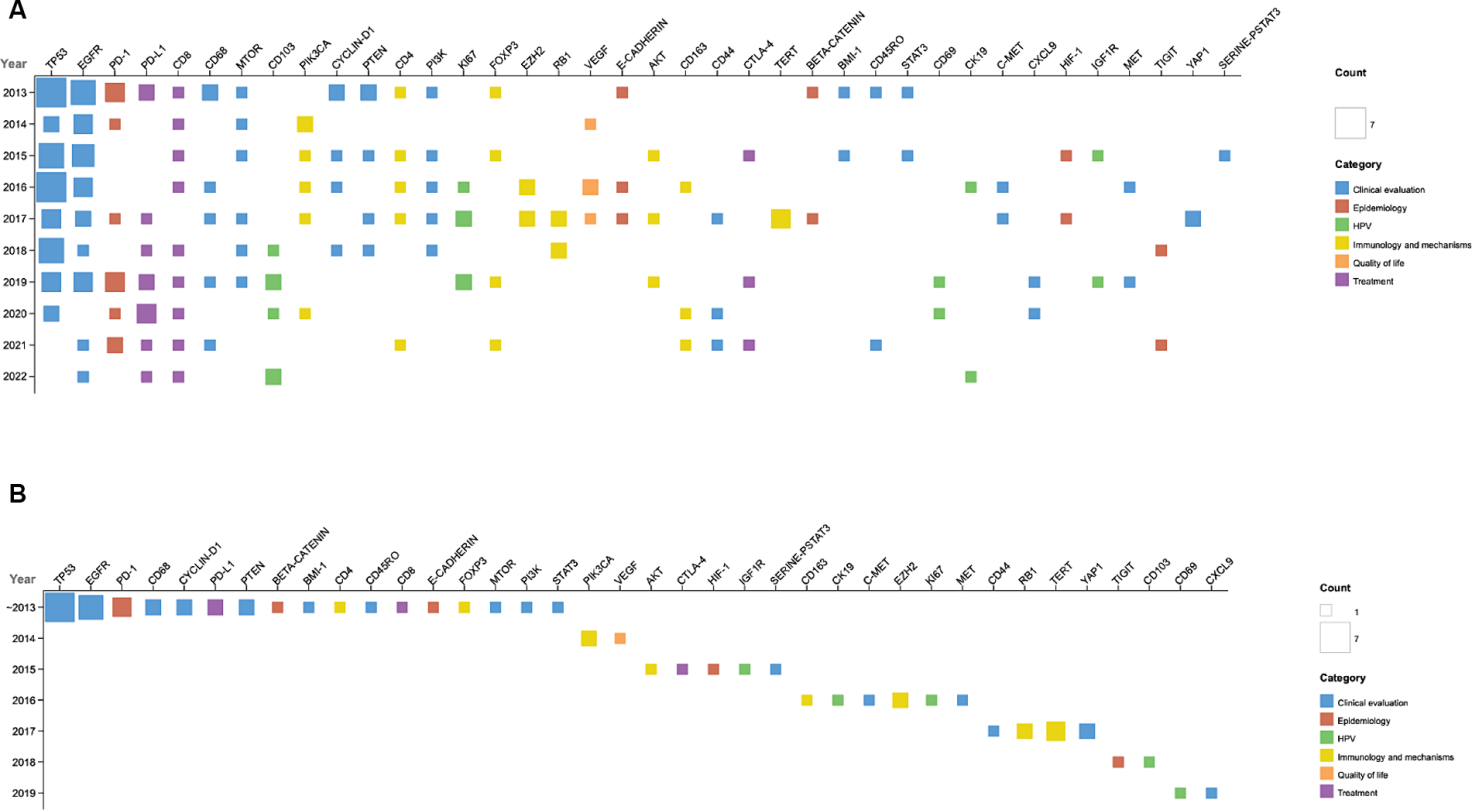
Gene analysis overview. **(A)** Yearly gene frequency. **(B)** Emerging genes: genes introduced in each year are displayed. ~2013: genes up to 2013.

Gene-based research is also notable. Figure 9A displays annual gene. TP53 and EGFR are most studied. TP53 encodes p53 protein and HPVE6 binding to p53 and promotes its degradation to exert its function (Nasman et al., 2020). Research on EGFR focuses on IgG1 monoclonal antibodies like cetuximab targeting EGFR.

Figure 9B illustrates the genes that emerged annually. Notably, in 2019, genes such as CD69 and CXCL9 were identified; both are closely linked to immune responses and cancer prognosis, suggesting new directions for research. CD69 is a classical marker of lymphocyte activation, influencing the differentiation of regulatory T cells and the secretion of IFN-γ, IL-17, and IL-22 (Cibrian and Sanchez-Madrid, 2017). A high presence of CD4+ CD69+ CD3+ T cells correlates strongly with a favorable prognosis in OPC (Hirna et al., 2021). Additionally, in the context of immunotherapy, an increased expression of CD69 in CD3+ T cells has been observed in responsive patients (Schumacher et al., 2024). CXCL9 is associated with favorable prognosis in solid tumors and positive responses to immunotherapy (Lieber et al., 2018; Han et al., 2019; Liang et al., 2021). In head and neck squamous cell carcinoma, CXCL9 enhances immune responses and infiltration, predicting the efficacy of immunotherapy (Huang et al., 2022).

## 4 Discussion

In this study, we conducted a bibliometric analysis and built a topic model for the literature on OPC. The aim of this research is to uncover the focus and dynamics of OPC studies, providing guidance for research hotspots and potential new research directions.

Bibliometric analysis indicated that Europe and America lead in this field, with significant contributions from their major research institutions. Keyword analysis shows a focus on epidemiology, diagnosis, and treatment, highlighting HPV as a central topic. The 2022 Keyword Burst reveals “American Joint Committee” and “guideline” as popular terms, possibly suggesting deep investigations based on HPV status. The reference timeline highlights “#16 disparities” as a current research focus, likely because the current stage of research aims to explore differences in survival and prognosis associated with HPV status.

Further topic modeling on influential articles confirmed that HPV (Topic 7) remains central. Our findings align closely with clinical practice, particularly in Europe and America, where there is a concentration of HPV-related OPC research. Additionally, tumor staging and stratification (Topic 9) and medical and social impacts (Topic 5) are gaining importance in recent years, strongly correlated with HPV. For instance, the AJCC8 staging incorporates HPV status as a key consideration (Amin et al., 2017), treating HPV-positive and -negative OPC distinctly, thus spurring staging and classification research. HPV identification has improved OPC prevention and mortality rates. HPV-positive patients have noticeably better clinical outcomes and can potentially be prevented through HPV vaccination (Marur et al., 2010; Lechner et al., 2022). Studies indicated that HPV vaccine reduces high-risk HPV oral infections, thereby lowering OPC incidence (Herrero et al., 2013; Chaturvedi et al., 2018; Katz, 2021).

Despite progress in preventing and diagnosing HPV-related OPC, challenges remain in screening, staging, and treatment. To better understand OPC research’s current state and future directions, we organized and summarized the unresolved challenges, guiding future studies.

### 4.1 Screening methods are not yet stable and reliable

Our results showed the key role of HPV (topic 7) in OPC. underscoring the need for HPV screening. However, OPC screening faces challenges unlike cervical cancer, due to oral saliva’s interference and unsatisfactory specificity (Nasman et al., 2020). Current techniques include testing for oral HPV DNA infection using mouthwash and gargle samples, collecting oropharyngeal cytology samples using brushes, detecting HPV-16 antibodies in blood, and testing for circulating HPV DNA, among others. Screening methods vary across studies. To date, no trials have yet randomly screened high-risk HPV-positive groups to assess health impacts, leaving no direct evidence that screening reduces morbidity or mortality risks (Day et al., 2020).

Compared to screening, current liquid biopsy research is increasingly focusing on blood circulating tumor DNA (ctDNA) for prognosis prediction. A 10-year follow-up study by Mazurek et al. (2024) demonstrated that pre-treatment ctHPV16 DNA viral load can be used to predict the risk of distant metastasis in HPV16 positive OPC patients. Studies on post-treatment ctDNA have shown that ctDNA within 12 weeks after treatment is a biomarker of minimal residual disease in head and neck squamous cell carcinoma and can predict disease progression (Honore et al., 2023). Notably, this study included both HPV-positive and HPV-negative patients, addressing the previous lack of data on HPV-negative patients.

### 4.2 The current staging system is still in need of improvement

HPV-positive OPC patients generally have a positive prognosis, spurring research in staging and stratification (Topic 3). Despite the current staging system accounting for HPV status, treatment guidelines need refinement. The latest AJCC8 staging shifts T1-T2N1 from stage III to stage I, yet evidence suggests these patients may benefit more from chemoradiotherapy instead of just radiotherapy or surgery (Cheraghlou et al., 2018; Kang et al., 2023). Notably, despite tumor location being a key prognostic factor, it’s not included in current AJCC staging guidelines. Additionally, incorporating factors like smoking history could enhance prediction comprehensiveness (Lechner et al., 2022).

### 4.3 New treatment methods require further data support

Head and neck squamous cell carcinoma (HNSCC) is one of the tumor types with the highest level of immune cell infiltration (Mandal et al., 2016). In recent years, with the deepening of understanding of the tumor immune microenvironment, immunotherapy has become a research hotspot in the field of tumor treatment. In this study, Topic 1 covers topics related to immune mechanisms and immunotherapy.

HPV-positive oropharyngeal cancer (HPV+ OPC) has different immune characteristics from HPV-negative oropharyngeal cancer (HPV- OPC). Compared with HPV- OPC, HPV+ OPC has a higher density of tumor-infiltrating lymphocytes (TILs), including CD3+ T cells, CD8+ T cells, Regulatory T cells (Treg cells), B cells, and plasma cells (Matlung et al., 2016; Gurin et al., 2020; Mito et al., 2021; Tosi et al., 2022). Among these, high levels of infiltrating CD20+ B cells and reduced macrophage infiltration are associated with a favorable patient prognosis (Wang Z. et al., 2023; Khoo et al., 2024). Tumor-associated macrophages can promote angiogenesis, nutrient deprivation, and immune suppression, thereby promoting tumor growth and treatment resistance. In addition, HPV+ OPC also expresses higher levels of PD-L1 (Tosi et al., 2022).

Therefore, we provided an overview of current research on immunotherapy. Considering the unique immune microenvironment and favorable prognosis of HPV+ OPC. We also explored the relationship between these strategies and HPV status, in order to reveal whether there are significant differences in treatment approaches for patients with different HPV statuses.

1. Immune checkpoint inhibitors and the impact of HPV status

Immune checkpoint inhibitors (ICIs) are an important class of drugs for the treatment of HNSCC. Pivotal clinical trials such as CHECKMATE-141 (Ferris et al., 2016), KEYNOTE-040 (Cohen et al., 2019), and KEYNOTE-048 (Burtness et al., 2019) have driven the application of ICIs in HNSCC treatment, gradually shifting them to first-line treatment options.

Based on this foundation, researchers have advanced their exploration into the diverse applications of immunotherapy, encompassing treatments post-resistance, as well as neoadjuvant and adjuvant therapies in the perioperative context. Specifically, regarding strategies for managing resistance to immunotherapy, the INTERLINK-1 study (Fayette et al., 2023b) evaluated the efficacy of monalizumab plus cetuximab in patients who progressed after treatment with ICIs. Unfortunately, this regimen did not improve patient survival. However, the study observed that patients in the cetuximab monotherapy group had better survival than historical data for patients who had not previously received immunotherapy, suggesting that immunotherapy may affect the sensitivity to subsequent treatment regimens. Therefore, further research is needed to identify treatment strategies for ICIs resistance in order to provide patients with more effective treatment options.

Meanwhile, researchers are also exploring the potential applications of ICIs in perioperative treatment. Compared to traditional postoperative radiotherapy and chemotherapy, the introduction of neoadjuvant and adjuvant immunotherapy has attracted widespread attention. For example, the NCT05522985 study (Wang H. et al., 2023) showed that neoadjuvant immunotherapy combined with chemotherapy can significantly improve the major pathological response (MPR) of HNSCC patients; the ADJORL1 trial (Guerlain et al., 2023) showed that nivolumab as adjuvant therapy after salvage surgery had better 2-year disease-free survival (DFS) and overall survival (OS) than reirradiation. These findings suggest that the application of immunotherapy in the perioperative period has demonstrated superior efficacy, potentially leading to significant changes in current standard treatment protocols in the future.

Although ICIs have made significant advances in the treatment of OPC, there are virtually no guidelines recommending the adjustment of immunotherapy regimens for OPC based on HPV status. This is due to the varying impacts of HPV status on the efficacy of ICIs, as shown by subgroup analyses in different trials. The CHECKMATE-141 study found that HPV-positive HNSCC patients benefited more, while the KEYNOTE-040 and INTERLINK-1 studies did not find an effect of HPV status on efficacy. In addition, many new clinical trials have also failed to clarify the relationship between HPV status and efficacy. In summary, current research on the relationship between HPV status and immunotherapy regimens lacks prospective analysis and sufficient sample sizes. The existing evidence is isolated and inadequate, not supporting definitive conclusions.

1. Applicability of Novel Immune Therapies

Currently, novel immunotherapies such as cancer vaccines have attracted widespread attention. Phase IIa study SAKK 11/16 evaluated the efficacy of MVX-ONCO-1 in patients with previously treated, recurrent/metastatic HNSCC (Mach et al., 2023). MVX-ONCO-1 is a novel cancer vaccine that combines autologous tumor cells with allogeneic cells containing a potent adjuvant, GM-CSF. The study results showed that MVX-ONCO-1 could significantly prolong the OS of patients with refractory recurrent/metastatic HNSCC. This therapy shows promising prospects, but further clinical trial data are needed to support its efficacy. Currently, there has been no subgroup analysis based on HPV status.

Due to the close association between HPV and OPC, researchers have shown significant interest in novel vaccine therapies for HPV-positive patients. For HPV-positive HNSCC patients, E6 and E7 proteins are ideal therapeutic targets as tumor-associated antigens (Chandra et al., 2017). MEDI0457 is a DNA vaccine composed of three plasmids expressing HPV-16/18 E6 and E7 oncoproteins and IL-12. A clinical trial evaluated the efficacy of MEDI0457 combined with durvalumab for HPV-16/18-related recurrent/metastatic HNSCC. While the primary efficacy endpoint of the study was not met, clinical responses were observed, with an objective response rate (ORR) of 27.6%, a median progression-free survival (PFS) of 3.5 months, and a median overall survival of 29.2 months (Aggarwal et al., 2023). In conclusion, initial research on cancer vaccines has demonstrated their therapeutic potential. However, further investigation into the relationship between HPV status and treatment outcomes, as well as the development of vaccines targeting HPV-positive patients, remains of high research value.

In addition to cancer vaccines, immunotherapies based on arenavirus are also starting to gain interest. A study evaluated the efficacy of HB-200 arenavirus-based immunotherapy combined with pembrolizumab for first-line treatment of HPV16-positive head and neck cancer patients. HB-200 can express non-oncogenic HPV16 E7-E6 fusion protein, inducing E6- and E7-specific CD8+ T cell responses (Lauterbach et al., 2021). The results showed that the combination therapy exhibited promising clinical activity in the first-line treatment setting, with an overall response rate of 43% (Nabell et al., 2023). It is worth noting that among the 20 patients included, 19 were patients with OPC, hence further results of this regimen in treating OPC are expected. This indicates the approach has potential in the treatment of OPC and warrants further exploration.

### 4.4 De-escalation therapy for HPV-positive OPC still requires confirmation

HPV+ OPC, distinct from HPV- OPC in clinical behavior and treatment response, generally has a better prognosis (Ang et al., 2014). Studies showed HPV-positive OPC’s greater radiosensitivity and improved treatment outcomes compared to HPV-negative cases (Fallai et al., 2009; Lassen et al., 2009; Chen et al., 2013). Thus, treatment side effects have gained attention, leading to a focus on “quality of life” and the frequent use of “Deglutition Disorders” and “Deglutition” as MeSH terms. Due to adverse reaction concerns, research on de-escalation treatment for HPV-positive OPC is increasing. However, despite extensive studies, results are still unsatisfactory, and current guidelines have not considered HPV status for de-escalation treatment criteria.

#### 4.4.1 Early surgical de-escalation: need for prospective data

The treatment options for early-stage OPC primarily include surgery and chemoradiotherapy. Retrospective analysis results show that for HPV-positive OPC, there is no significant difference in overall efficacy between the two methods (Wang et al., 2015). However, considering the potential long-term complications of chemoradiotherapy, surgical treatment may be a better choice for certain early-stage OPC patients, especially those who are HPV-positive (Parvathaneni et al., 2019).

Transoral robotic surgery (TORS) is a minimally invasive surgical technique widely used in the treatment of HPV+ OPC. TORS involves using a robotic system to access the patient’s mouth and excise affected tissue, serving as a de-escalation treatment strategy. Compared to traditional surgery, TORS offers better visualization of the surgical site, less trauma, and the ability to preserve the patient’s speech and swallowing functions (Sharma et al., 2016). It also offers higher cost-effectiveness compared to non-TORS surgeries (de Almeida et al., 2016; Motz et al., 2017). Studies have shown that TORS may result in a lower risk of postoperative dysphagia compared to chemoradiotherapy (More et al., 2013; Chen et al., 2015; Sethia et al., 2018). To avoid the long-term toxicity associated with multiple treatments, some countries recommend TORS as an alternative to primary radiotherapy for early-stage OPC (Williamson et al., 2023).

Although TORS is highly regarded as a treatment option for OPC, there are still studies expressing concerns about this approach. For example, a meta-analysis has shown that compared to chemoradiotherapy, TORS is associated with poorer swallowing function (Quan et al., 2021). Additionally, there is a lack of stratified research based on HPV status, and the current studies have small sample sizes. These factors indicate that the efficacy and safety of TORS in treating OPC still require further validation through more research. Therefore, more studies are needed to improve the efficacy and safety of TORS treatment for OPC and to clarify its value in different patient groups (De Virgilio et al., 2023).

#### 4.4.2 Radiotherapy de-escalation: need for level I evidence

Radiotherapy de-escalation for HPV-positive OPC aims to minimize side effects like swallowing difficulties while maintaining effectiveness (Gabani et al., 2019; Ma et al., 2019). This involves reducing radiation area and dose.

A phase 2 trial for stage 3–4 HPV-positive OPC showed 100% 5-year control and survival rates with 43.2Gy Elective Nodal Irradiation (ENI) and no irradiation of the contralateral oropharynx and level IV lymph nodes (Bahig et al., 2020). The OPTIMA trial also reported success in the de-escalation of radiotherapy for HPV-positive OPC (Seiwert et al., 2019; Rosenberg et al., 2021). This suggests that selective irradiation is a potentially effective de-escalation strategy, but specifics on its implementation still require further research data support.

For curative radiotherapy, de-escalation is typically based on staging, smoking history, or response to induction chemotherapy. Several phase 2 trials have confirmed that it’s feasible to implement radiotherapy de-escalation for p16-positive OPC patients based on staging and smoking history (Chera et al., 2018, 2019; Yom et al., 2021). In these trials, the radiotherapy dose was reduced from the standard 70Gy to 60Gy, but the use of cisplatin was different from the standard treatment, hence further studies are needed. Induction chemotherapy response-based de-escalation also shows promise (Chen et al., 2017; Marur et al., 2017; Seiwert et al., 2019; Rosenberg et al., 2021), although some argue this method does not genuinely lessen toxicity. Current guidelines propose radiotherapy de-escalation for patients with lower tumor burdens based on staging and other factors. Additionally, there is a lack of Level I evidence regarding de-escalation of radiotherapy in HPV-positive patients, and some study designs are insufficiently rigorous, necessitating more evidence for decision-making (Kang et al., 2023).

Overall, de-escalating radiotherapy for HPV-positive patients holds promise, but the potential risks must also be considered. Before deciding on de-escalated treatment, it is essential to carefully evaluate each patient’s specific circumstances and the risk–benefit ratio, avoiding decisions based solely on HPV status.

#### 4.4.3 Cisplatin: still in standard treatment

Cisplatin is crucial for advanced OPC treatment, but its severe side effects like emetogenicity, ototoxicity, and nephrotoxicity led to trials with cetuximab. Yet, cetuximab shows limited control over local and distant metastasis without reducing adverse reactions. It’s only an alternative for those unfit for cisplatin (Gillison et al., 2019; Mehanna et al., 2019).

The Phase II FRAIL-IMMUNE study evaluated the efficacy of durvalumab combined with paclitaxel and carboplatin as first-line treatment for patients with locally advanced or metastatic HNSCC who cannot tolerate cisplatin. The study results showed that this regimen met the primary endpoint, with a median OS of 18 months and a 12-month OS rate of 62.5%, demonstrating good anti-tumor activity and potentially becoming an effective treatment option for first-line HNSCC patients, but further confirmation in Phase III trials is needed. Additionally, the study did not observe any differences in overall survival among subgroups of OPC patients based on HPV status (Fayette et al., 2023a).

The HN002 trial studied omitting cisplatin in locally advanced p16-positive OPC treatment with IMRT, concluded that cisplatin is indispensable. Despite more acute AEs in combination therapy, grade 3–4 AE rates were consistent, and M. D. Anderson Dysphagia Inventory (MDADI) scores recovered within a year (Yom et al., 2021). Current research believes that even for HPV-positive patients, chemotherapy can still improve survival (Kang et al., 2023), thus maintaining cisplatin in standard treatment protocols.

### 4.5 Areas lacking attention

Our results reveal certain limitations: despite an increase in publications over the decade, the growth rate is modest, and no new MeSH terms have emerged recently. This indicates a lack of major breakthroughs in OPC research, with current studies delving deeper into existing findings. While HPV-positive OPC treatment outcomes are satisfactory, leading to research focus on prognosis and quality of life, other factors like smoking and poor oral hygiene are crucial OPC risks. In some regions, HPV-positive OPC is less common, and notably, HPV-negative OPC often has a worse prognosis and higher mortality. Therefore, HPV-negative OPC also deserves our adequate attention and research.

### 4.6 Future outlook

Our analysis underscores the role of HPV in OPC management, encompassing prevention, detection, treatment, and prognosis. Concerning prevention, about 100 countries promote the HPV vaccine, but mainly for women (Drolet et al., 2019). Vaccination rates remain low due to economic, socio-cultural and other constraints. In 2017, coverage among women aged 9–45 years was less than 2% (Harper and DeMars, 2017; Nasman et al., 2020). The higher incidence of HPV-positive OPC in men is concerning (Mahal et al., 2019), partly due to their more sexual partners and limited herd immunity from women’s vaccination (D'Souza et al., 2016). Therefore, there is evidence that men also hope to be protected by the vaccine (Grandahl et al., 2019). Increasingly, countries are adopting gender-neutral HPV vaccinations, a significant public health advancement. For HPV detection, p16 immunohistochemistry is common, with high sensitivity (94%) and specificity (83%) (Prigge et al., 2017). Future efforts will focus on developing cost-effective, rapid, and accurate HPV diagnostics, possibly using CRISPR technology (Kellner et al., 2019; Deutsch et al., 2022). Regarding treatment, while current guidelines do not fully integrate HPV status into de-escalation standards, research in this area shows promise. Our goal is to enhance treatment efficacy while reducing side effects, moving toward more personalized approaches.

### 4.7 Limitations

Our study has limitations. It only includes English papers from WOSCC from the last decade, possibly missing studies in other databases or languages. Also, the LDA algorithm may add subjectivity in topic naming and categorization. Still, our research provides clinicians with a quick understanding of key topics in the field.

## 5 Conclusion

In our study, we employed bibliometrics and topic modeling to analyze key OPC research topics from 2013 to 2022, including HPV, epidemiology, treatment, and more. While HPV research emphasizes treatment de-escalation for better patient quality of life due to its positive prognosis, a shift in treatment standards requires more evidence. Meanwhile, the less-favorable prognosis and under-researched HPV-negative OPC demand more exploration.

## Data availability statement

The original contributions presented in the study are included in the article/Supplementary material, further inquiries can be directed to the corresponding authors.

## Author contributions

ZL: Data curation, Writing – original draft. HWa: Data curation, Writing – original draft. YX: Writing – original draft. HWe: Writing – original draft. YZ: Project administration, Writing – review & editing. HD: Project administration, Writing – review & editing.

## Supplementary material

FIGURE S1 Number of topics determined using ldatuning in KH Coder. The optimal topic count is suggested when the upper two lines are at their lowest while the bottom two lines peak.
