# Supplementary figures and images for: Oropharyngeal cancer and human papillomavirus: a visualization based on bibliometric analysis and topic modeling
Source: Front Microbiol. 2024 May 29;15:1387679. doi: 10.3389/fmicb.2024.1387679 (PMC11197978; doi:10.3389/fmicb.2024.1387679)

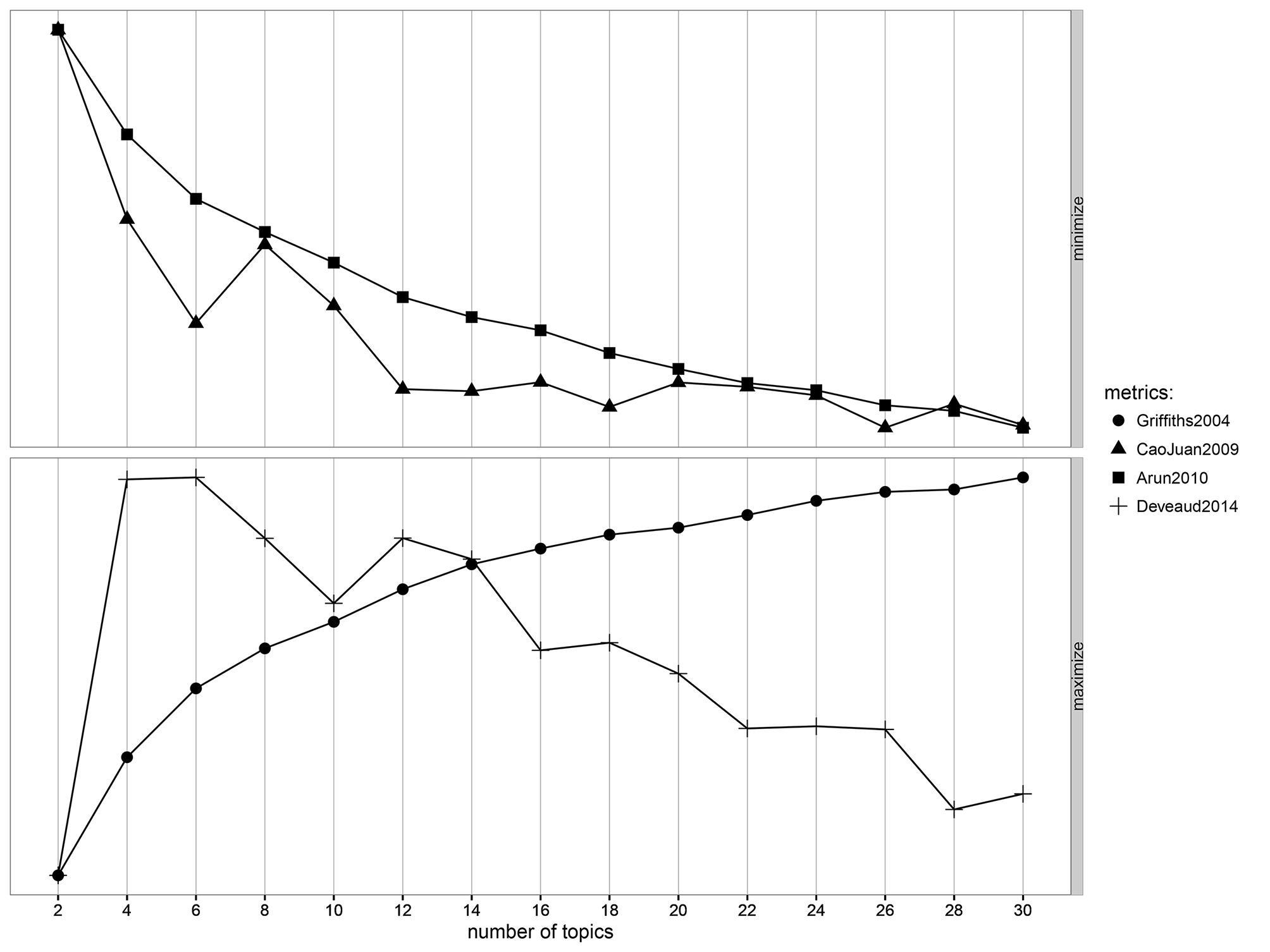

Supplement: FIGURE S1 — Number of topics determined using ldatuning in KH Coder. The optimal topic count is suggested when the upper two lines are at their lowest while the bottom two lines peak. [file Image_1.TIF]
