# Supplementary material for: Oropharyngeal cancer and human papillomavirus: a visualization based on bibliometric analysis and topic modeling
Source: Front Microbiol. 2024 May 29;15:1387679. doi: 10.3389/fmicb.2024.1387679 (PMC11197978; doi:10.3389/fmicb.2024.1387679)
Supplement: Supplementary file 2 [file Table_1.docx]

**Supplementary Table 1 Search strategy**

Bibliometric Analysis

| #1 | TS=(oropharyngeal carcinoma* OR oropharyngeal neoplasm* OR oropharyngeal cancer* OR tonsil cancer* OR tonsil carcinoma* OR tonsil neoplasm* OR base of tongue cancer* OR base of tongue carcinoma* OR base of tongue neoplasm* OR soft palate carcinoma* OR soft palate neoplasm* OR soft palate cancer*) |
| --- | --- |
| #2 | FPY=(2013-2022) |
| #3 | LA=(English) |
| #4 | DT=(Article) |
| #5 | #1 AND #2 AND #3 AND #4 |

A total of 7355 publications were involved

Topic Modeling

| #1 | TS=(oropharyngeal carcinoma* OR oropharyngeal neoplasm* OR oropharyngeal cancer* OR tonsil cancer* OR tonsil carcinoma* OR tonsil neoplasm* OR base of tongue cancer* OR base of tongue carcinoma* OR base of tongue neoplasm* OR soft palate carcinoma* OR soft palate neoplasm* OR soft palate cancer*) |
| --- | --- |
| #2 | FPY=(2013-2022) |
| #3 | LA=(English) |
| #4 | DT=(Article) |
| #5 | #1 AND #2 AND #3 AND #4 |
| #6 | IS=( 0007-9235 OR 1471-0072 OR 0028-4793 OR 1474-1776 OR 0140-6736 OR 1759-4782 OR 1474-175X OR 1740-1526 OR 0098-7484 OR 1087-0156 OR 1078-8956 OR 1471-0056 OR 1474-1733 OR 2056-676X OR 0028-0836 OR 0036-8075 OR 1759-5045 OR 0732-183X OR 1474-4422 OR 1759-5029 OR 1759-4758 OR 0092-8674 OR 1470-2045 OR 0959-535X OR 2159-8274 OR 1061-4036 OR 1471-003X OR 0923-7534 OR 1759-5002 OR 2213-8587 OR 2374-2437 OR 1074-7613 OR 1535-6108 OR 2213-2600 OR 0195-668X OR 0009-7322 OR 1465-7392 OR 1548-7091 OR 0732-0582 OR 1759-5061 OR 1476-4598 OR 1550-4131 OR 2214-109X OR 0893-8512 OR 2001-3078 OR 1001-0602 OR 1529-2908 OR 0031-6997 OR 0003-4819 OR 0168-8278 OR 1473-3099 OR 1097-6256 OR 1934-5909 OR 2589-7500 OR 0735-1097 OR 0066-4154 OR 0006-4971 OR 1553-4006 OR 0017-5749 OR 0016-5085 OR 2168-6106 OR 2468-2667 OR 1552-5260 OR 1931-3128 OR 0962-8924 OR 1750-984X OR 1759-4790 OR 1560-2745 OR 1364-6613 OR 0302-2838 OR 0163-769X OR 0167-7799 OR 0066-4278 OR 0149-5992 OR 0003-4967 OR 2352-3026 OR 1368-7646 OR 2468-1253 OR 2168-6149 OR 1097-2765 OR 2058-5276 OR 2470-9468 OR 0169-5347 OR 0270-9139 OR 1756-8722 OR 0009-7330 OR 2095-5138 OR 0896-6273 OR 0966-842X OR 0305-1048 OR 0066-4197 OR 2198-3844 OR 1471-4906 OR 0903-1936 OR 0162-8828 OR 0737-4038 OR 2168-6203 OR 1554-8627 OR 1936-0851 OR 1350-9047 OR 1044-579X OR 1556-0864 OR 1388-9842 OR 2522-5839 OR 0066-4227 OR 0169-409X OR 1545-9993 OR 1433-7851 OR 2041-1723 OR 2405-8025 OR 2051-1426 OR 0027-8874 OR 0006-8950 OR 1754-2189 OR 0105-4538 OR 0105-2896 OR 0003-4932 OR 0008-5472 OR 1078-0432 OR 1522-8517 OR 0305-7372 OR 1540-1405 OR 2235-1795 OR 1672-7681 OR 0887-6924 OR 2001-1326 OR 1542-3565 OR 0027-8424 OR 1756-9966 OR 2326-6066 OR 1549-1277 OR 2044-5385 OR 2056-5968 OR 2168-6181 OR 0300-0729 OR 0194-5998 OR 2042-6976 OR 1916-0216 OR 1043-3074 OR 0378-5955 OR 0196-0202 OR 2331-2165 OR 1976-8710 OR 1525-3961 OR 0937-4477 OR 0023-852X OR 0196-0709 OR 0179-051X OR 1749-4478 OR 1879-7296 OR 1531-7129 OR 0392-100X OR 2378-8038 OR 1808-8694) |
| #7 | #5 AND #6 |

A total of 1681 publications were involved
